# Depression and Anxiety Changes Among Sexual and Gender Minority People Coinciding with Onset of COVID-19 Pandemic

**DOI:** 10.1007/s11606-020-05970-4

**Published:** 2020-06-17

**Authors:** Annesa Flentje, Juno Obedin-Maliver, Micah E. Lubensky, Zubin Dastur, Torsten Neilands, Mitchell R. Lunn

**Affiliations:** 1 Community Health Systems, University of California, San Francisco, San Francisco, CA USA; 2 Alliance Health Project, Department of Psychiatry, School of Medicine, University of California, San Francisco, CA USA; 3 The PRIDE Study/PRIDEnet, Stanford University School of Medicine, Stanford, CA USA; 4 Department of Obstetrics and Gynecology, Stanford University School of Medicine, Stanford, CA USA; 5 Center for AIDS Prevention Studies, Department of Medicine, School of Medicine, University of California, San Francisco, CA USA; 6 Division of Nephrology, Department of Medicine, Stanford University School of Medicine, Stanford, CA USA

## INTRODUCTION

With SARS-COV-2 recently sweeping the globe, the population is experiencing a group stressor unlike any phenomenon in this country in the last century. How the pandemic experience is related to mental health challenges including anxiety and depression is unknown. Numerous factors—such as changes in community function; restriction of activities and social contacts; and fearfulness about the virus, the economic downturn, and food access—may contribute to poorer mental health. Marginalized populations, such as sexual and gender minority people (i.e., non-heterosexual people and transgender or gender-expansive people, respectively) may be particularly at risk for adverse impacts of the pandemic due to preexisting economic and health factors.^1^ We set out to document changes in depression and anxiety within The PRIDE Study, a longitudinal cohort of sexual and gender minority people, a vulnerable population.^2^

## METHODS

Participants in The PRIDE Study, a longitudinal cohort study of sexual and gender minority people,^2^ were included if they completed mental health measures in the 2019 Annual Questionnaire (timepoint 1, June 2019—ongoing at time of data extraction) and in a COVID-19 impact ancillary study (timepoint 2, March 23, 2020, through April 19, 2020). Paired sample *t* tests examined changes in depression (9-item Patient Health Questionnaire, PHQ-9^3^) and anxiety (7-item Generalized Anxiety Disorder Scale, GAD-7^4^) symptoms overall and separately among those who screened positive (PHQ-9 and GAD-7 scores ≥ 10^3, 4^) and negative (scores < 10) for depression and generalized anxiety disorder at timepoint 1.

## RESULTS

In total, 2288 participants were included in this study (see Table 1 for participant characteristics). Depression symptoms increased by a mean PHQ-9 score of 1.21 (*t*[2280] = 11.35, *p* < .001, *d =* .20) from timepoint 1 to 2. Anxiety symptoms increased by a mean GAD-7 score of 3.11 (*t*[2282] = 27.95, *p* < .001, *d =* .54). Among individuals who screened positive for depression at timepoint 1, PHQ-9 scores decreased by a mean of 1.08 (*t*[670] = − 4.80, *p* < .001, *d =* .21) at timepoint 2. Among individuals who screened negative for depression at time 1, PHQ-9 scores increased by a mean of 2.17 (*t*[1609] = 19.58, *p* < .001, *d =* 0.53) at timepoint 2. Among individuals who screened positive for generalized anxiety at timepoint 1, there was no change in GAD-7 scores (*t*[508] = 1.01, *p* = .32, *d =* .06). Among individuals who screened negative for generalized anxiety at timepoint 1, GAD-7 scores increased by a mean of 3.93 (*t*[1773] = 32.93, *p* < .001, *d =* .88) at timepoint 2.

**Table 1:** Demographic characteristics of *N =* 2288 sexual and gender minority individuals

Participant characteristics	
Age: mean, median (SD)	36.9, 31.9 (14.7)
Race/ethnicity,^a^ *n* (%)	
American Indian/Alaska Native	65 (2.8%)
Asian	98 (4.3%)
Black/African American	78 (3.4%)
Hispanic, Latino, or Spanish	128 (5.6%)
Middle Eastern or North African	28 (1.2%)
Native Hawaiian or Pacific Islander	7 (0.3%)
White	2116 (92.5%)
Another race or ethnicity	26 (1.1%)
Sexual orientation,^a^ *n* (%)	
Asexual	268 (11.7%)
Bisexual	693 (30.3%)
Gay	834 (36.5%)
Lesbian	467 (20.4%)
Pansexual	320 (14.0%)
Queer	923 (40.3%)
Questioning	56 (2.4%)
Same-gender loving	97 (4.2%)
Straight/heterosexual	39 (1.7%)
Two-spirit	13 (0.6%)
Another sexual orientation	86 (3.8%)
Gender,^a,b^ *n* (%)	
Agender	99 (4.3%)
Cisgender man^c^	418 (18.3%)
Cisgender woman^c^	623 (27.2%)
Genderqueer	300 (13.1%)
Man	562 (24.6%)
Non-binary	438 (19.1%)
Questioning	85 (3.7%)
Transgender man	279 (12.2%)
Transgender woman	124 (5.4%)
Two-spirit	23 (1.0%)
Woman	500 (21.9%)
Another gender identity	134 (5.9%)
Sex assigned to individual at birth, *n* (%)	
Female	1428 (63.0%)
Male	840 (37.0%)
Highest level of education, *n* (%)	
Less than high school completion	20 (0.9%)
High school diploma or equiv.	487 (21.3%)
College degree (2- or 4-year)	885 (38.7%)
Graduate degree	895 (39.1%)
Income, *n* (%)	
< $20,000	797 (35.2%)
$20,000–60,000	801 (35.4%)
$60,000–100,000	369 (16.3%)
$100,000+	296 (13.1%)
Mental health: mean, median (SD)	
PHQ-9 timepoint 1	7.10, 6 (5.99)
PHQ-9 timepoint 2	8.31, 7 (6.43)
GAD-7 timepoint 1	5.78, 4 (5.21)
GAD-7 timepoint 2	8.89, 8 (6.22)

^a^Individuals could select more than one option; thus, categories are not mutually exclusive

^b^This includes people who were assigned a sex of birth of male or female, and only gender is reported here; thus, gender minority people may be found in all categories

^c^Cisgender is listed here as an identity label. Cisgender people can be found in multiple categories and may not endorse this identity label

## DISCUSSION

We found increases in anxiety and depression coinciding with the COVID-19 pandemic onset. Increased anxiety and depression symptoms were driven by people who did not have preexisting symptoms consistent with generalized anxiety or depression. While this study was conducted with sexual and gender minority people, the results may be relevant for other vulnerable populations, such as other minority groups.

Health care providers are advised to check in with patients about stress and to screen for mood and anxiety disorders, even among patients who had no prior history of anxiety or depression. Treatment and referrals can include traditional interventions such as individual therapy and medications and may also include COVID-19-specific supports implemented on a larger scale (e.g., supportive peer-led groups, mindfulness practice). This study is observational. Our finding that individuals with preexisting depression had improved mood from timepoint 1 to 2 may represent regression to the mean and should not be interpreted that these individuals have less depressive symptoms, as they already were experiencing symptoms of depression at timepoint 1. Future research will identify who is most at risk for adverse impact. In the interim, we should consider ways to support the mental health of all of our communities during the pandemic, with special care and attention to vulnerable populations.
